# Diagnostic Approaches to Adult-Type Diffuse Glial Tumors: Comparative Literature and Clinical Practice Study

**DOI:** 10.3390/curroncol30090568

**Published:** 2023-08-24

**Authors:** Vincentas Veikutis, Mindaugas Brazdziunas, Evaldas Keleras, Algidas Basevicius, Andrei Grib, Darijus Skaudickas, Saulius Lukosevicius

**Affiliations:** 1 Medical Academy, Lithuanian University of Health Sciences, LT50161 Kaunas, Lithuania; mindaugas.brazdziunas@gmail.com (M.B.); evaldas.keleras@lsmu.lt (E.K.); algidas.basevicius@kaunoklinikos.lt (A.B.); darijusskaudickas@gmail.com (D.S.); saulius.lukosevicius@lsmuni.lt (S.L.); 2 Faculty of Medicine, Kaunas University of Applied Sciences, LT44162 Kaunas, Lithuania; 3 Department of Internal Medicine, Nicolae Testemitanu State University of Medicine and Pharmacy, MD2004 Chisinau, Moldova; andreigrib@yahoo.com

**Keywords:** glial tumors, diagnostic approaches

## Abstract

Gliomas are the most frequent intrinsic central nervous system tumors. The new 2021 WHO Classification of Central Nervous System Tumors brought significant changes into the classification of gliomas, that underline the role of molecular diagnostics, with the adult-type diffuse glial tumors now identified primarily by their biomarkers rather than histology. The status of the isocitrate dehydrogenase (IDH) 1 or 2 describes tumors at their molecular level and together with the presence or absence of 1p/19q codeletion are the most important biomarkers used for the classification of adult-type diffuse glial tumors. In recent years terminology has also changed. IDH-mutant, as previously known, is diagnostically used as astrocytoma and IDH-wildtype is used as glioblastoma. A comprehensive understanding of these tumors not only gives patients a more proper treatment and better prognosis but also highlights new difficulties. MR imaging is of the utmost importance for diagnosing and supervising the response to treatment. By monitoring the tumor on followup exams better results can be achieved. Correlations are seen between tumor diagnostic and clinical manifestation and surgical administration, followup care, oncologic treatment, and outcomes. Minimal resection site use of functional imaging (fMRI) and diffusion tensor imaging (DTI) have become indispensable tools in invasive treatment. Perfusion imaging provides insightful information about the vascularity of the tumor, spectroscopy shows metabolic activity, and nuclear medicine imaging displays tumor metabolism. To accommodate better treatment the differentiation of pseudoprogression, pseudoresponse, or radiation necrosis is needed. In this report, we present a literature review of diagnostics of gliomas, the differences in their imaging features, and our radiology’s departments accumulated experience concerning gliomas.

## 1. Introduction

The expansion of knowledge in the central nervous system (CNS) tumor’s molecular alterations has been massive in the last decade. Former tumors have been defined histologically. Molecular information only provided complementary data [1]. However, despite having equivalent histological patterns, treatment outcomes for IDH-wildtype and IDH-mutant diffuse gliomas were substantially different [2].

Molecular diagnostics in recent years have changed not only clinical outcomes but also the whole classification of glial tumors. The most recent alterations were made in 2021. The biggest changes include the distinctive features that are seen in adults and children. Gliomas and other neuronal tumors are divided into six finer groups, but adult-type diffuse gliomas are the most relevant in clinical practice. Adult-type diffuse gliomas now are identified primarily by their biomarkers rather than histology. This family includes three types of tumors: astrocytoma (IDH-mutant), oligodendroglioma (IDH-mutant, and 1p/19q-codeleted), and glioblastoma (IDH-wildtype). All diffuse adult-type astrocytomas, IDH-mutants, are considered a single type and are graded as 2, 3, or 4; oligodendroglioma, IDH-mutant, and 1p/19q-codeleted are graded as 2, 3; and glioblastomas comprise only IDH-wildtype tumors and are graded as 4. Therefore, being IDH-wildtype tumors, glioblastomas are now a separate diagnosis from astrocytomas, IDH-mutant tumors. However, for a astrocytic glioma to be able to qualify as glioblastoma the tumor should at least have microvascular proliferation, a site of necrosis, mutation of the TERT promoter, EGFR gene amplification, or changes in the copy number of +7/−10 chromosomes [3].

A contemporary approach to preoperative diagnosis, better patient care, and post-treatment imaging set new perspectives for glial tumor diagnostics [4]. This article aims to evaluate the literature about the radiological approach to diagnostics of adult-type glioma imaging as it is one of the most fatal outcomes for CNS tumors in adults and compare our department’s experience, containing over 200 cases of adult-type diffuse glioma patients.

## 2. Biomolecular Diagnostics

Conventionally histological verification for CNS tumor grading is needed for diagnosis. The advances in molecular markers have implemented them in diagnostics and treatment decision making, including findings of ATRX, TP53, or CDKN2A/B signals of astrocytoma [3]. In the following paragraphs, we will be discussing the most relevant markers.

### 2.1. Isocitrate Dehydrogenase (IDH)

IDH mutations are considered important in glioma genesis, determining a more favorable outcome and longer survival with mutated IDH, than patients with wild-type IDH; therefore, IDH-1 status can also be used as a clinical prognosis indicator [5,6,7,8]. The presence of IDH mutations excludes glioblastomas according to the 2021 Classification of CNS Tumors, as all glioblastomas are IDH-wildtype.

By combining radiological features with genetic signatures, a wider view of glial tumors can be achieved [9]. A study of 280 patients who were diagnosed with glioblastoma and underwent surgical treatment showed that tumor contrast enhancement [10], multifocality [11], tumor location [12,13], edema [11], and cysts [14] can be linked with genetic attributes and survival outcome in glioblastoma patients. In our hospital, patients are routinely checked for IDH when a glial tumor is suspected. IDH-1 mutations are identified by using DNA pyrosequencing [15,16]. *A* link between IDH-1 and contrast accumulation in the site of the lesion, cysts or locating in the frontal cortex, vibrant margins, and homogenous signals are observed equivalent data to be found in other publications also.

However, it is important to make decisions based on collective knowledge combined with radiographic imaging. For example, tumor-induced edema could be used to predict the survival outcomes of glioblastoma patients based on *MGMT* promoter methylation, not the IDH-1 status [8]. Genetic alterations provide biological data on the tumor, supplementing radiological imaging, and, hence, more accurate treatment decisions [17]. Nevertheless, a IDH-mutant variant tumor does not always ensure a better outcome [18].

### 2.2. 1p19q Codeletion

1p19q codeletion together with IDH mutation decides the course of treatment for oligodendroglioma. It is an evident biomarker concerning long-term survival after aggressive multimodal treatment [19]. Such courses can be considered by combining surgical resection, followed by radiotherapy and chemotherapy with procarbazine, CCNU (lomustine), and vincristine (PCV) [20]. IDH-mutant and 1p/19q codeleted grade-3 oligodendrogliomas have a dramatically longer overall survival median when treated with radiotherapy and PCV in comparison to only radiotherapy treatment. In contrast, survival rates are significantly shorter for patients with 1p/19q intact grade-3 gliomas. No statistically meaningful (*p* > 0.05) disparity was observed comparing survival rates for radio-chemotherapy and radiotherapy treatments. By testing patients for 1p/19q codeletion not only a more accurate classification can be achieved but it also has a clinical meaning, and more precise treatment can be adapted [21].

### 2.3. MGMT Promoter

O (6)-methylguanine-DNA methyltransferase (MGMT) is a DNA repair protein that can neutralize its alkylation when chemotherapy is administered. Hypermethylation of the *MGMT* promoter results in gene silencing [22,23]; therefore, gliomas with methylated MGMT promoters are more susceptible to the effects of alkylating agent therapy, such as temozolomide. In our hospital, the evaluation of MGMT promoter status is performed for all glioma patients when chemotherapy with temozolomide is considered. MGMT has been the biomarker with the most meaningful influence in clinical decision making for resilient glioblastomas since its discovery [24]. Patients with methylated tumors with little or no edema have particularly longer survival [8]. While MGMT promoter methylation predicts a more favorable treatment response, in some malignant glioma patients treated with radiotherapy in combination with temozolomide it is shown to be associated with pseudoprogression, a pathological feature that can imitate true tumor progression on followup diagnostic imaging. Imaging features were found to poorly predict MGMT promoter methylation in one study [8].

## 3. Imaging Techniques for the Guidance of Glioma Diagnostic

### 3.1. Computed Tomography

While most glial tumors can be diagnosed on computed tomography, it is a less comprehensive imaging modality when compared to MRI; therefore, it plays a secondary role in the diagnostic imaging of gliomas. As a routine in our clinical hospital, it is used for immediate postoperative followups to check for possible bleeding or other complications. CT imaging is sensitive enough for long-term posttreatment tracking. However, if tumor progression has been detected, the patient should be directed for MRI [25]. CT can also be used as the main imaging modality on rare occasions when MRI is contraindicated (ferromagnetic foreign bodies, pacemakers, and cochlear implants are most common).

### 3.2. MRI

By having intricate and subtle architectural changes in the brain, magnetic resonance imaging (MRI) is sensitive enough to suspect the radiographical characteristics of glioma. The usage is not instrumental in making a diagnosis but also in pre- and posttreatment [26].

#### Standard Imaging Sequences

In recent years more advanced imaging has entered oncological diagnostics; however, basic MRI sequences are still the foundation of the radiological workload. They show the location, size, margins, structure, and spread of the tumor, and the presence or absence of vasogenic edema [27,28,29]. T1 contrast-enhanced (T1CE) images with gadolinium-based contrast agents reveal disruption of the blood–brain barrier. Susceptibility-weighted images (SWI) are helpful for a better depiction of tumoral hemorrhages and calcifications. Diffusion-weighted images can show areas of increased diffusion, while automatically calculated apparent diffusion coefficient (ADC) may have a role in predicting the tumor grade for gliomas and evaluating posttreatment response.

Oligodendroglioma usually appears as cortical and subcortical white matter mass, that can be heterogeneous due to cystic degeneration, calcifications, or small intratumoral hemorrhages with mild or absent contrast enhancement or peritumoral edema. Slow growth is a typical followup image sign.

Low-grade diffuse astrocytoma usually presents as a homogeneous relatively well-marginated white-matter mass without necrosis or contrast enhancement, with mild or absent peritumoral edema, and typically shows slow growth.

The typical appearance of glioblastoma is a white-matter-centered mass with a central necrotic core, surrounded by thick irregularly enhanced margins and surrounded by vasogenic edema. Glioblastoma is the most common and aggressive type of malignant brain tumor in adults [9,30]. Overall, the prognosis is poor, with a median survival of <2 years [31,32], the same clinical outcomes also seen in our hospital. However, an early and precise diagnostic is key to better treatment and, therefore, longer survival. 

Despite the advancements in biomolecular diagnostics that can be beneficial for a better outcome for brain tumors, they also bring new challenges to diagnostic imaging. The radiologic appearance of the tumor may not entirely correspond to its biomarkers.

**Figure 1:**
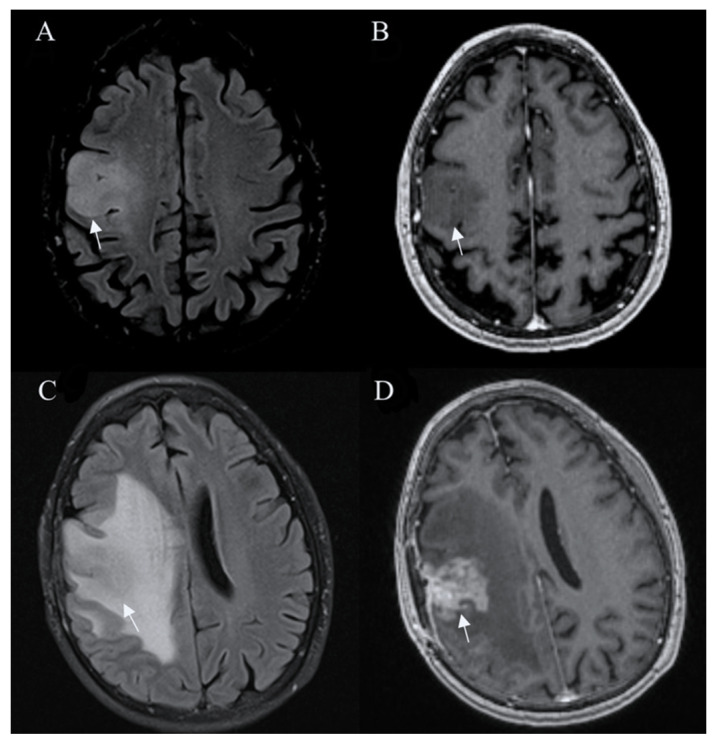
Glioblastoma IDH wild-type. Axial T2-FLAIR (**A**) and postcontrast axial (**B**) T1W images are suggestive of a low-grade tumor. However, followup FLAIR (**C**) and postcontrast T1W (**D**) images 6 months later show tumor progression with irregular contrast enhancement, surrounded by extensive edema, characteristic of glioblastoma. Arrows in A-D images shows the mass-like site of IDH wild-type glioblastoma. Images used for publication are taken from LUHS Radiology Clinic archives servers.

Tumors with IDH-wildtype usually show aggressive growth and transformation, even when they initially present with radiologic features of low-grade glioma (Figure 1).

**Figure 2:**
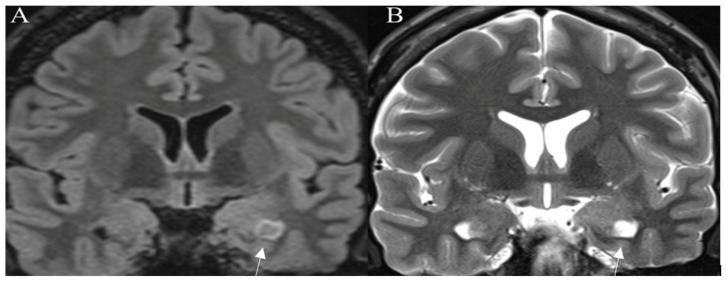
T2-FLAIR mismatch sign. Histologically proven IDH mutant, 1p19q nondeleted astrocytoma. (**A**) Coronal T2-FLAIR image showing an area of lower intensity surrounded by a hyperintense peripheral rim (arrow). On the corresponding (**B**) coronal T2W image an area of the homogenously hyperintense signal is seen (arrow). Images used for publication are taken from LUHS Radiology Clinic archives servers.

A T2-FLAIR mismatch sign is considered a highly specific imaging biomarker for IDH-mutant, 1p/19q-non-codeleted diffuse glioma, with T2w sequence showing well-circumscribed high-intensity mass, that appears relatively hypointense and usually with a hyperintense rim on T2-FLAIR images (Figure 2).

*MGMT* promoter methylated glioblastoma tends to reveal limited peritumoral edema, high ADC values, and low CBV [33].

In glioblastomas with a substantial proportion of noncontrast-enhancing tumors, a mass-like pattern in most cases correlates with longer survival [18].

When the volume of the lesion, involvement of the cortex, whether the high or low-grade tumor is suspected, and genetic profile are taken into consideration, sometimes it can be difficult to formulate a correct decision. In most cases, sequences such as FLAIR, T1*, and T2* are sufficient for suspecting a glial neoplasm. However, a tendency for advanced imaging is increasing [18,34,35].

### 3.3. Perfusion-Based Imaging

Perfusion-weighted imaging provides spatial blood flow through tissue. Due to signal changes in glial tumor blood flow circulation, a more profound conclusion can be made. A healthy tissue can maintain metabolism, remove byproducts, and keep a stable temperature; however, pathological tissue cannot sustain these processes. Mainly two approaches are used for MR perfusion. Dynamic susceptibility contrast (DSC), which heavily depends on contrast uptake (mostly gadolinium-chelate) and dynamic contrast enhancement (DCE), is another contrast-related sequence that depends on the imaging approach when perfusion defects and hyperdense regions of the lesion are seen. The last one (arterial spin labeling (ASL) does not require contrast media; instead, advanced rapid pulse sequences and blood flow “act” as a contrast [36].

#### 3.3.1. Dynamic Susceptibility Contrast

**Figure 3:**
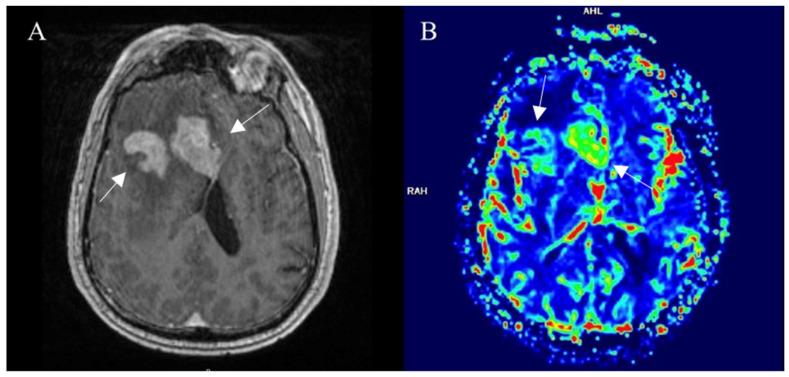
Right frontal lobe glioblastoma, pretreatment imaging. (**A**) Postcontrast axial T1W image shows a contrast-enhancing part of a tumor (arrows), that displays elevated perfusion on the DSC-based cerebral blood volume map (arrows) (**B**). Images used for publication are taken from LUHS Radiology Clinic archives servers.

DSC is more useful when discussing cerebral tissue. Due to having a large vessel network and, thus, by contrast remaining in the blood flow system. The paramagnetic nature of the contrast agent increases local tissue susceptibility, causing increased T2∗ dephasing of nearby tissues. Gradient echo and well-perfused tissue exhibit a reduction in the signal relative to the precontrast images or the poorly perfused tissues. This criterion is a substitute marker for capillary density or neoangiogenesis and often is relative to the contralesion brain tissue. The duration and reliability of DSC are the main advantages. However, calculations of absolute parameter measures and sensitivity to susceptibility-related artifacts depend on the user. Artifacts are commonly observed at the base of the skull or the site of postoperative hemosiderin deposition [37]. Tumor growth leads to neovascularity in high-grade gliomas; therefore, microvascular density is increased which leads to elevated relative cerebral blood volume (rCBV). For such patients, DSC may be helpful in the preoperative diagnosis (Figure 3) or followup of malignant lesions [38]. It is the most common technique of brain perfusion. In our radiology department, we use it routinely for posttreatment followup of high-grade gliomas.

#### 3.3.2. Dynamic Contrast Enhancement

DCE is a T1-weighted sequence that usually uses the spoiled gradient echo technique; therefore, longer effectuation (fulfilment) time is required compared with DSC [39]. When imaging is obscure due to microvascular permeability or the blood–brain barrier, DCE gains an advantage against other perfusion-related techniques. Also, compared with DSC, reduction in susceptibility-related artifacts has been reported [40]. Disadvantages include the longer scan time, decreased temporal resolution, and disagreements about the best suitable contrast substance. Even though DCE has a decreased temporal imaging capability, when a lesion with mixed pathology is discovered it is still the preferred method due to improved spatial resolution [36]. Hence, the preferred modality is based on the tumor’s localization; other factors taken into consideration can include the nature of the tumor and its vascularity.

#### 3.3.3. Arterial Spin Labelling

Lastly, when discussing perfusion adaption for glial tumors arterial spin labelling (ASL) uses different sets of images than DSC or DCE. Mainly, two technique alterations are being used for ASL [41]. Signals for both methods are primarily being made due to moving spins of blood and no statistical difference for diagnostics has been seen [42]. However, ASL has some setbacks. The main one is that long scanning times are needed, and motion artefacts cannot be evaded. Also, this modality is heavily dependent on the radiographer and the complexity of flow calculations means that ASL is not used in everyday clinical workflow [43]. Because of these listed reasons, the use of ASL is more historic and as other articles state; we also use it very occasionally in our clinical practice.

### 3.4. Advanced MR Imaging

#### 3.4.1. Spectroscopy

In most cases, MRI provides all the needed information about the tumor size and its tissue extension. However, sometimes the information can be inconclusive for pseudoresponse or pseudoprogression evaluation [44]. Even though functional and molecular imaging can provide more accurate information and lately these methods attracted a lot of attention, getting data about lesions metabolism is sometimes also needed. Magnetic resonance spectroscopy imaging (MRS) is a technique that provides metabolomic information despite overlayed anatomical structures [45].

**Figure 4:**
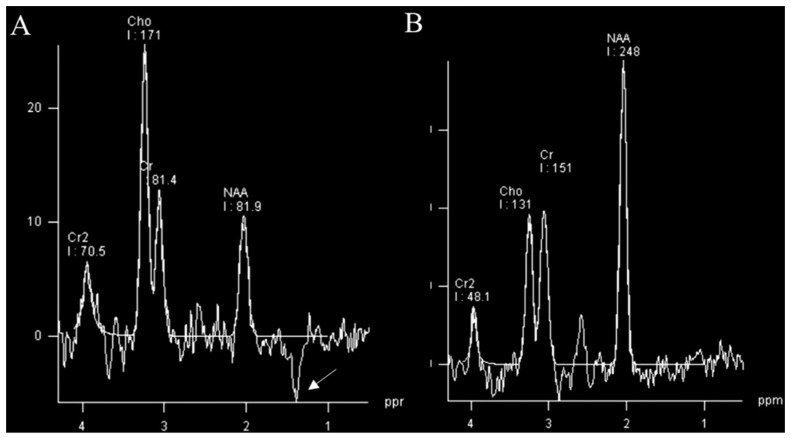
MR spectroscopy (TE 135 ms) of glioblastoma (**A**) and normal control (**B**). MRS of glioblastoma shows a raised choline (Cho) peak, a depressed N-acetyl aspartate (NAA) peak, and an inverted lactate peak (arrow) at 1.3 ppm. Images used for publication are taken from LUHS Radiology Clinic archives servers.

A high percentage of brain tumors have decayed signals for N-acetyl aspartate (NAA). The changes in neuronal tissue: temperature, metabolism, and byproducts exchange lead also to increased levels of Choline (Cho). It is observed that glioblastomas are linked with peaked Cho levels in the lesion. Knowing that glioblastomas often have the site of necrosis in which anaerobic oxidation overtakes the energy production, leading to increased levels of lactate explaining Cho [46]. There is a direct correlation between lactate levels and glioma grade [47] (Figure 4). Increased lipid levels are believed to be due to necrosis and membrane breakdown and are usually present in high-grade neoplasms and often absent in low-grade gliomas. A high myoinositol peak is more characteristic of lower-grade neoplasms [48].

Because of new advances in molecular diagnostics of brain tumors, it has been observed that 2-hydroxyglutarate (2-HG) can be a promising biomarker. More specifically, a subtle change during the glioma genesis IDH mutation occurs that starts producing 2-hydroxyglutarates. More than 99% of all IDH-mutated cells exhibit risen 2-HG levels. Taking into consideration the fact that there has been no background of healthy brain tissue with 2-HG, makes the marker a good indicator of glioma [49]. MRS is not only useful in detecting glioma but also has advantages in tumor evaluation for treatment response [50].

MR spectroscopy can be a useful tool for posttreatment followup. After radiotherapy or chemotherapy, if no change or elevated Cho peak is observed, relapse or progression should be taken into consideration [46].

MRS has not been widely accepted as a routine clinical tool for tumor evaluation; in our hospital, it is used only occasionally. Relatively low sensitivity, especially for the detection of low-concentration metabolites, and additional time are needed for examination to limit the application of MRS. However, combined with other imaging it can give insightful information about tumor metabolism and helps with diagnosis correction.

#### 3.4.2. fMRI

**Figure 5:**
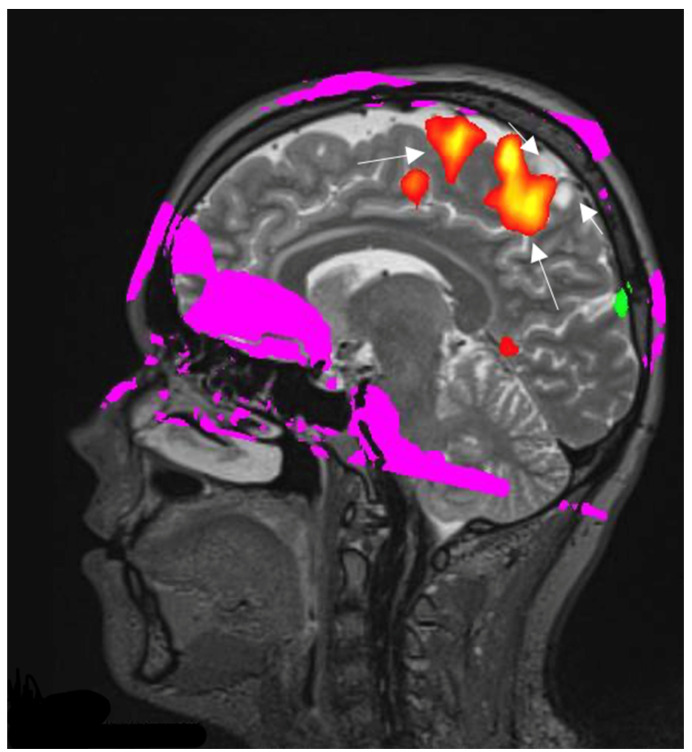
Right hemisphere astrocytoma. BOLD fMRI data are superimposed on sagittal T2W images for anatomic localization. BOLD fMRI showing the foot sensorimotor cortex (long arrows) is located along the anteroinferior aspect of the tumor (short arrows). The image used for publication is taken from LUHS Radiology Clinic archives servers.

For decades physicians struggled with neurological assessment concerning various senses. In some cases, for example, a glial tumor can interfere with motor and sensory functions of a patient, and functional MRI (fMRI) and a better understanding. This modality is based on the basic principles of MRI physics. Endogenous oxygenated hemoglobin is diamagnetic and has increased signal waves in comparison to deoxygenated hemoglobin, which has a shorter relaxation time on T2* resulting in a decreased signal. By applying these assumptions brain activation can be monitored. Stimulated cortex areas will have increased blood flow with higher levels of oxygenated blood. Naturally, deoxygenated levels of hemoglobin are reduced in comparison. It also minimizes the susceptibility of the cortex by dephasing induced signals on the T2* compared to unstimulated tissue. It all leads to a higher signal on T2*-weighted imaging. This phenomenon is described as the blood-oxygenation-level-dependent effect or simply BOLD (Figure 5) [51]. When discussing mainly glioblastomas, fMRI has quite an impact on surgical treatment planning. It gives a wider perspective of what the prognosis the patient could have and if it is necessary at all [52].

#### 3.4.3. Diffusion Tensor Imaging

DTI is a method that reconstructs a model of subcortical connectivity. Doing so can help more accurately plan the resection site based on the tract’s invasion level. It is also more likely to reduce the functional impairments postsurgically, helping neurosurgeons to be aware of the location of white matter tracts. Glioma’s heterogenic nature makes it difficult to differentiate from normal tissue and, thus, DTI would be helpful to segregate the two. Recent studies suggest that DTI is more efficacious when combined with other modalities. Combined with a tumor-isolating “fence-post” catheter (insertion of catheters around the border of tumor margins) technique, motor-evoked potentials from cortical areas can facilitate the resection of high-grade glioma up to 1 cm from the corticospinal tract [49]. 

**Figure 6:**
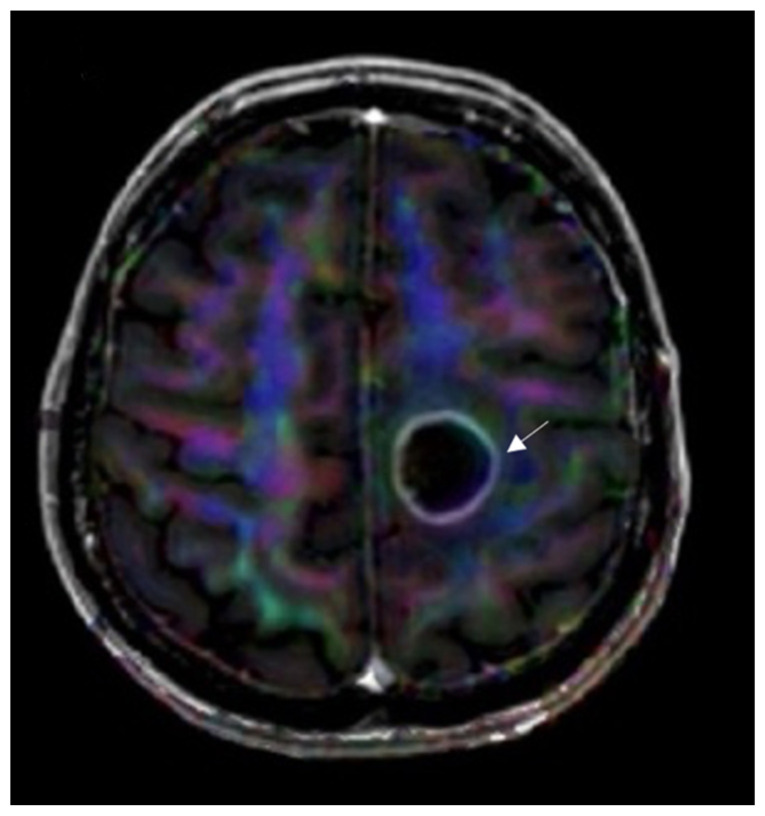
DTI image data superimposed on axial T1W postcontrast image for anatomic localization. Invasion of the left corticospinal tract is seen by a left frontal glioblastoma (arrow). The image used for publication is taken from LUHS Radiology Clinic archives servers.

Further advancements in diffusion sequencing have allowed finer imaging which initiated a neuronal fiber to be reconstructed [53]. A decision in resection site concerning white matter has made not only neurosurgeon planning more advanced but also ensures finer gliomas treatment [54,55]. Using high-definition fiber tractography it became possible to evaluate perilesional white matter tracts in case of glial tumors [56]. However, tractography heavily relies on the performing physician’s competence. The quality of an image depends on the regions of interest (ROIs) and the visualized tracts segmentation. The accuracy and sensitivity of fiber tracking algorithms can be analyzed using intraoperative electrical stimulation [57]. The imaging method suffers from the need to adjust the parameters, particularly to patient datasets, and even regions of interest. The lack of unified algorithm standardization makes tractography less approachable. Other problems include reliance on user interaction, placement of the seeds and mismatches between ROIs inclusion or exclusion, and a lack of image noise reduction. Therefore, a false positive or false negative tract’s pathological visualization can occur. However, tractography provides a depiction of global connectivity. Most importantly, the main technique’s advantage is the capability of tracts noninvasive 3D visualization that gives hope for future patients [57,58]. In LUHS DTI and white matter, tractography is performed for surgery planning when tumoral involvement of major white matter tracts is expected (Figure 6).

### 3.5. Nuclear Medicine Imaging

#### 3.5.1. Positron Emission Tomography

Positron emission tomography (PET) is paving the way in understanding complex heterogenous tumors such as gliomas. The glioma genesis is still not understood completely; however, including PET gives a better understanding of the tumor’s genesis. One of the hardest aspects of posttreatment diagnostics is the complexity and ability to remodel. It is one of the main reasons why it is hard to discern TP from PsP or radiation necrosis. By having a better comprehension of the tumor’s ecosystem, a better prognosis and treatment plan could be possible [59]. One of the most promising trackers that has been seen is 18F-FDG. Utilizing it for a recurrent glioma could have a better treatment prognosis. Amino acid PET is starting to become a standard when a PET scan is needed. The basis is simple, labeled amino acids can detect tumors progression in the earliest stages and, if so, a different approach could be thought of and improvements in prognosis could happen. As seen, PET is an advanced diagnostic tool for refining prognosis [60].

#### 3.5.2. SPECT

**Figure 7:**
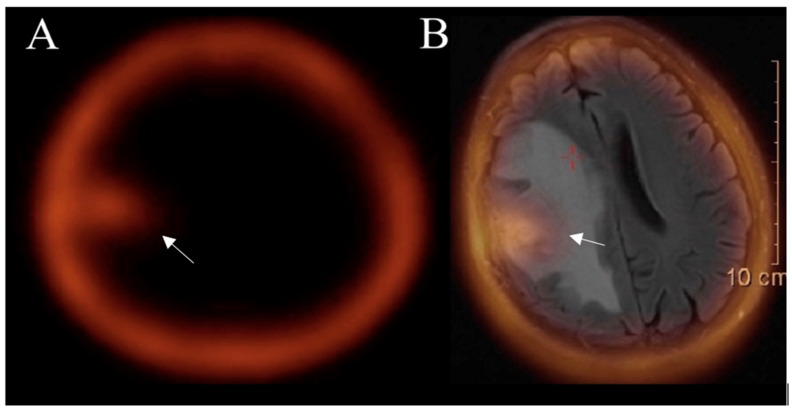
SPECT (**A**) and SPECT-MRI-fused (**B**) images show an accumulation of 99mTc- MIBI in a tumor area (arrow) after treatment, confirming the true progression of glioblastoma. Images used for publication are taken from LUHS Radiology Clinic archives servers.

When comparing nuclear medicine imaging SPECT is not only more widely accessible but also cheaper than PET; however, due to the attribution of nuclear decay, gamma rays used (two for PET, one for SPECT) makes the spatial resolution inferior compared to PET. However, isotopes used for SPECT are sensitive enough to observe various process regarding glial tumors. Technetium-99m-labelled compounds have been one of the main tracers used for differentiation between glioma progression and radiation necrosis. When 99mTc-sestamibi and 99mTc-tetrofosmin enter the blood flow no signs of conversion in a healthy brain have been registered. If uptake is seen, tumors recurrence or necrosis induced by radiation can be expected. Hence, the assimilation of radioactive tracer can be a differentiative tool. Other studies have shown that the cutoff ratio value of the tumor’s uptake for true progression is around 4; however, for tumors necrosis uptake varies but never peaks as high as for true progression [61]. One study discovered that 99mTc-tetrofosmin SPECT has the same level of accuracy as perfusion MRI in detecting recurrent tumors after glioma treatment [62]. In our clinical practice, SPECT 99mTc-sestamibi is used as an additional method to differentiate true progression from radiation necrosis, when conventional and advanced MRI provide doubtful results (Figure 7).

The diagnostic value of 99mTc-methionine SPECT is like PET FDG utility and higher than contrast-enhanced MRI for the detection of glioma recurrence [63]. A meta-analysis that assessed the efficacy of SPECT in distinguishing between glioma recurrence and radiation necrosis reported extremely high specificity and sensitivity [64].

### 3.6. Posttreatment Imaging

#### 3.6.1. True Progression

**Figure 8:**
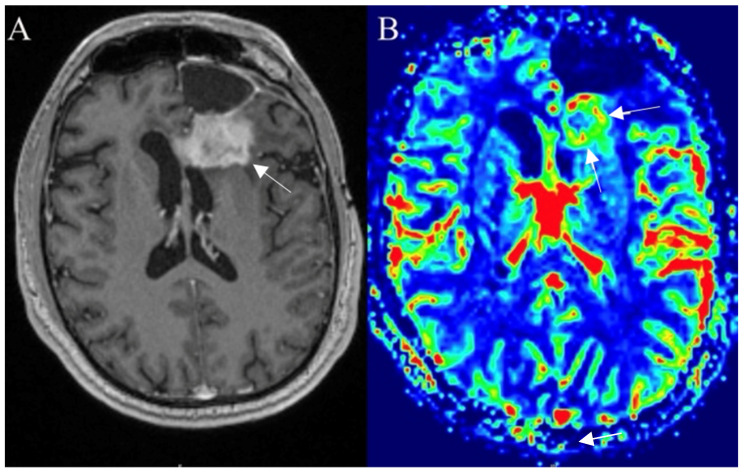
Left frontal glioblastoma showing progression after surgery, radiotherapy, and chemotherapy. Postcontrast axial T1W image (**A**) reveals an enhancing mass with elevated perfusion at the location of contrast enhancement (arrows) on DSC–based cerebral blood volume (CBV) map (arrows) (**B**). Images used for publication are taken from LUHS Radiology Clinic archives servers.

Criteria defining progression was first introduced in 2010. The RANO (Response Assessment in Neuro-oncology) guidelines depict true progression concerning imaging features also reflecting on chemoradiation time of completion (<12 weeks and >12 weeks). Enhancement outside of the radiation field, enlargement of perpendicular diameter by 25% or greater between the first and twelves week postradiotherapy scan, or clinical deterioration were progression-determining factors. The guidelines additionally considered the influence made on the imaging of antiangiogenic drugs. Increased FLAIR signal for nonenhancing lesions in such patients could indicate disease progression [65]. Improvements in immunotherapy treatment forced modulating RANO guidelines which adjusted the time frame for imaging criteria. The interval broadens to less than 6 months and more than 6 months after the start of immunotherapy. The timing was given to observe if the condition is getting worse while the immune response spreads. In 2017, modified RANO criteria focused on the differentiation between true progression and pseudoprogression based on at least two images taken in a month (Figure 8).

#### 3.6.2. Pseudoprogression

Post-treatment radiographic changes can be challenging for a clinician on excluding tumor progression (TP) from pseudoprogression (PsP). In a study of 208 patients, PsP is observed relatively commonly. A correlation was observed between MGMT-methylated tumors and PsP. Converting to numbers, 31% of the sample group was diagnosed with PsP. A more sophisticated treatment planning should be carried out dealing with methylated tumors if a preferable outcome is to be expected. Hence, PsP patients can endure a more robust treatment, achieving longer progression-free survival compared to TP patients [66]. Although, radiologic assessment in neuro-oncology has come a long way since McDonald’s criteria, which only had four basic features and relied solely on MRI. It is still frequently a tough decision to pass in the clinical field when it comes to distinguishing TP/PsP [67]. In situations such as radiation-induced treatment basic MRI imaging cannot always segregate the differences between PsP and TP. At first imitation of progression in the early stages of healing can occur, relying only on conventional imaging can be troublesome. In the application of chemoradiation, a common appearance of glioblastoma can be present on FLAIR, impeding diagnostics. Hence, advanced imaging is indispensable in contemporary glial tumor assessment. However, combining advanced imaging techniques such as DWI, PWI, or MRS has a better chance of differentiating TP from PsP [40]. DSC perfusion in PsP usually shows reduced cerebral blood volume, while viable tumors will usually have increased rCBV [68]. MRS in PsP reveals a low Cho and Cho/NAA ratio ≤1.4, while due to cell death, ADC values are expected to be elevated, with mean values ≥1300 × 10^−6^ mm^2^/s [69].

**Figure 9:**
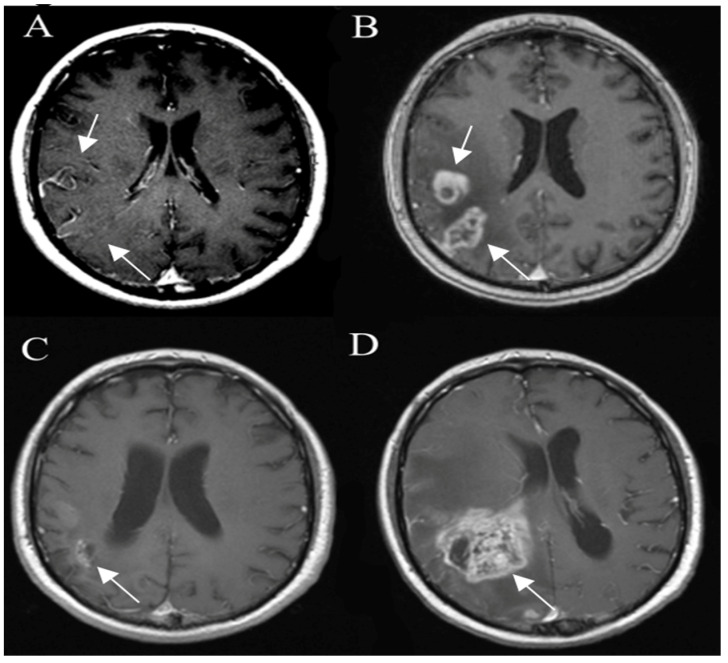
Glioma pseudo progression and true progression, postcontrast axial T1W images. Glioma (**A**) is shown as an ill-defined area of the tumor (arrow), with no significant contrast enhancement. A followup image (**B**) 3 months later shows the development of areas of irregular contrast enhancement (arrow) and surrounding edema due to pseudoprogression. The next followup 9 months later (**C**) shows contrast-enhancing areas (arrow) resolving without new treatment. However, 3 months later (**D**) there is a new rapidly growing heterogeneous enhancing lesion (arrow) due to true progression. Images used for publication are taken from LUHS Radiology Clinic archives servers.

It is also observed that IDH mutation frequently is detected in patients who have pseudoprogression (Figure 9). In comparison, most of the time the corpus callosum is involved in tumor progression, particularly in combination with the multiple enhancing lesions crossing the midline and spreading in the subependymal regions [70]. To correctly evaluate the patient’s survival and to make a correct clinical decision, it is of paramount importance to differentiate PsP from TP [71]. In our clinical practice, we use DSC perfusion and DWI as a part of the routine examination to differentiate between true progression and pseudoprogression of glial tumors or radiation necrosis, and SPECT is added in more difficult cases when MRI is not sufficient.

#### 3.6.3. Pseudoresponse

Another significant post-treatment highlight worth discussing is a pseudoresponse. The main difference from PsP is that pseudoresponse is observed in the setting of antiangiogenic therapy. VEGF, hepatocyte growth factor, fibroblast growth factor, platelet-derived growth factor, angiopoietins, and IL-8 are the proangiogenic agents known for glioblastoma’s angiogenic growth upregulation. Brain tumors express the VEGF-A factor in a considerable huge amount. Bevacizumab is a humanized monoclonal antibody that normalizes tumor vascularization by decreasing vessel size and permeability [31]. Therefore, tumor exposure to chemotherapy and/or radiation therapy is improved significantly. Response rates differ between 25% and 60% [72]. Changes in radiological features are followed as early as one day after initiation of anti-VEGF therapy [72]. Imaging findings show decreased contrast enhancement, edema, and vessel permeability. Sadly, even having this kind of impact, no correlation between bevacizumab and prolonged survival has been proven [73]. However, patients show a longer timeframe for progression-free survival and the need for steroid treatment. Nevertheless, patients who show a response radiographically develop a rapid worsening of the disease. The changes are best seen in no-enhancing T2 signal hyperintensity on T2 FLAIR sequences [74]. In the mind of pseudoresponse evaluation, the ADC value is not as informative for determining pseudoprogression or true progression. A recent study of ADC values exhibited normalization of the values following the administration of the bevacizumab [75]. Nevertheless, for improved overall survival prediction of recurrent glioblastoma patients, ADC values greater than 1.24 μm^2^/ms can be beneficial [76].

#### 3.6.4. Radiation Necrosis

Even though radiation necrosis is the opposite extreme of pseudoprogression, several studies refer to it as a single collective entity. However, pseudoprogression and radiation necrosis are diverse from each other in timing, pathological mechanisms, histopathology, and prognosis [77,78]. Pseudoprogression typically occurs up until a few months after treatment, whereas radiation necrosis can be seen after a prolonged time, typically between nine to twelve months but there have been cases when it was observed even after several years. This peculiarity occurs because new areas of contrast enhancement are bounded by the initial radiation field [79]. When it comes to survival prognosis, pseudoprogression has a more favorable outcome compared with radiation necrosis. Life quality is also affected by radiation necrosis, as neurologic functions often decline [80]. Patients with a 1p/19q codeletion can expect a much higher risk of developing radiation necrosis compared with other genetic markers [81].

**Figure 10:**
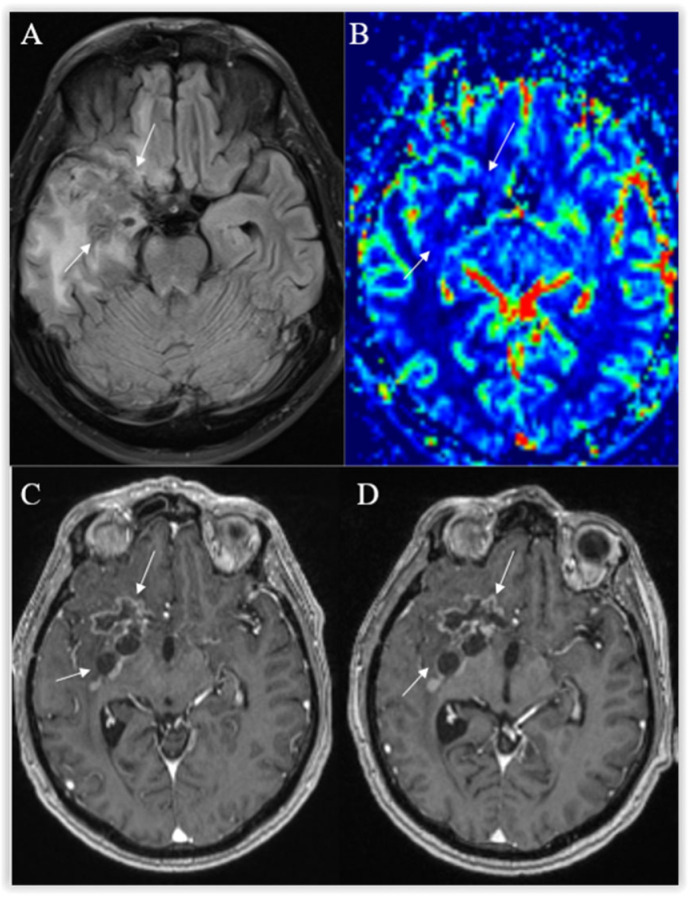
Radiation necrosis. Axial T2-FLAIR (**A**) and postcontrast axial T1W (**B**) images show heterogeneous an irregularly shaped area with cavitations, surrounded by moderate peripheral rim enhancement (arrows) and a wide T2-hyperintense zone of radiation-induced encephalopathy, with no significant mass effect. No frank hyperperfusion is seen on the DSC-based cerebral blood volume map (**C**). There is no significant change on a followup postcontrast axial T1W image (**D**) 11 months later.

The distinctive features of radiation necrosis are associated with feeble circulation within the periventricular white matter or contiguous fields to the radiation affected by radiation necrosis are seen. Occurrences in contralesioned sites or multifocal distribution were also reported [79]. “Swiss cheese” or “soap-bubble” images are more exhibited in the presence of radiation necrosis. These are internal enhancement patterns, and the margins are described as “feathery” in the peripheral or diffusive “mesh-like enhancement” pattern [82]. Central necrosis when compared to the lesion’s solid part will appear as a hyperdense signal on T2-weighted imaging [83]. The recurrent tumor typically has lower ADC values than radiation necrosis. Perfusion MR reveals decreased rCBV in areas of radiation necrosis (Figure 10). MRS shows elevated lactate/lipid peak and marked reduction of NAA, choline, and creatine. To differentiate between radiation necrosis and tumor progression, we always include DWI and MR perfusion in the post-treatment followup MRI examination, with the occasional use of MR spectroscopy, and SPECT is added if necessary.

#### 3.6.5. Imaging after Immunotherapy

Immunotherapy has come a long way since it was introduced. In 2018 Nobel Prize in Medicine was awarded to James Allison and Tasuku Honjo for their breakthrough research in immunotherapy. Today, it is mostly used as an adjuvant treatment; however, it is believed that immunotherapy is the future of cancer treatment [84]. In the setting of treatment, an increase in lesions can reflect a localized inflammatory response despite immunotherapy. A new, enhancing lesion may be the response of the immune system in previously nonenhancing, infiltrative disease. “Flare phenomenon” or overdue response can occur [85]. Some researchers investigated the changes seen in perfusion and MRS after immune treatment [86,87]. A lipid peak may be seen, as lipids are a substrate of natural killer (T cells) in the setting of immunotherapy response. According to our experience, it is important to check the medical history comprehensively for patients undergoing immunotherapy, to avoid hyperdiagnostic for glioma patients. Another aspect that needs to be considered is the glioma immune tumor microenvironment (TME). Nevertheless, the treatment has an expectancy of promising results; however, in the TME area glioma-associated macrophages, myeloid-derived suppressor cells, and brain-resident cells compose obstacles to the treatment [88]. Since the breakthrough of immunotherapy, there have not been conventional guidelines for the response evaluation and so the immunotherapy response assessment in neuro-oncology (iRANO) criteria was developed. The main differences from RANO criteria are as follows: within 6 months after the start of immunotherapy, the appearance of the new lesions without significant clinical decline should not automatically be interpreted as a progressive disease; to confirm disease progression a repeat scan is needed 3 months or later.

## 4. Conclusions

Glial tumors are among the most malignant brain tumors. New research in the biomolecular field helps to differentiate and better foresee the glioma’s outcome, which is reflected in a new 2021 WHO Classification of CNS Tumors. While standard diagnostic imaging usually provides necessary information for identifying and characterizing adult-type diffuse brain gliomas, advanced imaging techniques such as fMRI and DTI may be required for treatment planning. However, differentiation between true progression, pseudoprogression, and radiation necrosis on posttreatment followup imaging can be challenging and usually additionally requires perfusion MRI as part of a routine protocol on followup examination. When tumor progression is suspected, MR spectroscopy and SPECT or PET imaging can be of value when the result of the routine examination remains ambiguous. For the best glial tumor treatment results, a multimodal approach is needed. Combining various imaging techniques, and considering the strengths and limitations, the radiologist can develop a more evidence-based assessment.

## Data Availability

No new data were made in our review article.
